# Curcumin and saroglitazar attenuate diet-induced nonalcoholic steatohepatitis by activating the Nrf2 pathway and suppressing ERK1/2 signaling

**DOI:** 10.22038/IJBMS.2024.75293.16320

**Published:** 2024

**Authors:** Reza Afarin, Negar Dinarvand, Hossein Azizi Dariuni, Ghazal Orak, Bahar Jaberian Asl, Reza Azizi, Azam Khedri

**Affiliations:** 1 Cellular and Molecular Research Center, Medical Basic Sciences Research Institute, Ahvaz Jundishapur University of Medical Sciences, Ahvaz, Iran; 2 Hyperlipidemia Research Center, Ahvaz Jundishapur University of Medical Sciences, Ahvaz, Iran; 3 Department of Basic and Laboratory Sciences, Khomein University of Medical Sciences, Khomein, Iran

**Keywords:** Curcumin, ERK1/2, Non-alcoholic fatty liver - disease (NAFLD), Nrf2, Saroglitazar

## Abstract

**Objective(s):**

Non-alcoholic fatty liver disease (NAFLD) is a chronic steatohepatitis disorder. If left untreated, it can progress to hepatocellular carcinoma. Several studies have shown that saroglitazar, a PPARα/γ dual agonist, and curcumin (the principal constituent of turmeric) may be effective in the treatment of NAFLD. This research aimed to study the pharmacological mechanism of these compounds in rats with NAFLD.

**Materials and Methods:**

NAFLD was induced in male Wistar rats (aged 6–8 weeks) by feeding them a high-fat diet (HFD) for 6 weeks. Subsequently, the rats were divided into four groups, with Group 1 continuing on HFD, while groups 2, 3, and 4 received HFD supplemented with saroglitazar, curcumin, and both saroglitazar and curcumin, respectively. We evaluated the expression of Nrf2, ERK1/2, NOX1,2,4, antioxidant enzymes, PPARα, γ, and genes regulating lipid metabolism in the liver. Histopathology of liver tissue was also examined. Furthermore, we analyzed serum levels of lipid profiles and hepatic enzymes.

**Results:**

Rats with NAFLD that received treatment involving saroglitazar and curcumin showed a significant decrease in the expression of ERK1/2, SREBP1, PPARγ, pro-inflammatory cytokines, NOXs, and ROS levels. Additionally, the levels of Nrf2, PPARα, and antioxidant enzymes showed a significant increase. The serum levels of lipid profiles and hepatic enzymes also decreased significantly after drug treatment.

**Conclusion:**

Our results confirm that both saroglitazar and curcumin ameliorate NAFLD by regulating the Nrf2 and ERK1/2 signaling pathways. These findings suggest that curcumin could serve as a suitable substitute for saroglitazar, although they appear to have a synergistic effect.

## Introduction

Non-alcoholic fatty liver disease (NAFLD) is a progressive chronic liver disorder worldwide (1, 2), resulting from the accumulation of lipids in more than 5% of hepatocytes without secondary causes, such as consumption of alcohol or drugs (3). NAFLD affects over 25% of the world’s population (4). While NAFLD itself may not cause direct damage to liver cells (3), it can progress to advanced stages, including non-alcoholic steatohepatitis (NASH), fibrosis, cirrhosis, and potentially liver cancer (5, 6). 

Although the biochemical processes underlying the formation of NAFLD remain unclear (7), it has been suggested that this condition is intricately related to metabolic syndrome (4). When the synthesis or uptake of fatty acids exceeds the capacity for β-oxidation, it leads to accumulation of triglycerides within the liver, known as steatosis, a characteristic feature of NAFLD. This condition is associated with increased beta-oxidation of fatty acids and enhanced electron flow through the mitochondrial electron transport chain, contributing to increased reactive oxygen species (ROS) production (8). Excess ROS contribute to oxidative stress (OS) and inflammation, both recognized as pivotal factors in the initiation and progression of NAFLD (7).

The family of peroxisome proliferator-activated receptors (PPARs), comprising three known isotypes (α, β/δ, and γ, each with distinct functions and tissue distribution), is a group of ligand-activated transcription factors that regulate various biological functions, including lipid metabolism, insulin sensitivity, oxidative stress, and inflammation. For example, PPARs suppress the level of pro-inflammatory factors including tumor necrosis factor-alpha (TNF-α), transforming growth factor-beta (TGF-β1), interleukin-1 (IL-1), and interleukin-6 (IL-6) (9, 10). These physiological processes are factors associated with NAFLD (11). Therefore, PPARs appear to be attractive targets for the prevention or treatment of NAFLD, NASH, dyslipidemia, etc. (12).

PPARγ up-regulates genes involved in lipid biosynthesis, while PPARα reduces fatty acids synthesis through repressing sterol regulatory element-binding protein 1 (SREBP1) expression and enhances fatty acid beta-oxidation through up-regulation of carnitine palmitoyltransferase 1a (CPT-1a) (13, 14). SREBP1, a transcription factor with two isoforms, SREBP1a and SREBP1c, plays a crucial role in fatty acid metabolism by activating key enzymes such as fatty acid synthase (FAS), acetyl-CoA carboxylase (ACC), etc. (15). Furthermore, SREBP-1 also stimulates the expression of PPARγ (16).

 On the other hand, there is a positive feedback loop between PPARγ and Nuclear factor (erythroid-derived 2)-like 2 (Nrf2) because several studies have revealed that PPARγ is a target gene of Nrf2 and also enhances its activation (17). Nrf2, functioning as a transcription factor, up-regulates the production of antioxidant factors like superoxide dismutase (SOD), glutathione peroxidase (GPx), heme oxygenase-1 (HO-1), and others, while simultaneously suppressing the expression of NADPH oxidases (NOX). (18, 19). The NOX family produces O2-free radicals and consists of seven isoforms, with NOX 1, 2, and 4 being the primary isoforms in liver tissue (20). Consequently, enhancing the function of Nrf2 serves to mitigate oxidative damage (21). SOD converts O2− into H_2_O_2_ and oxygen (O2), and subsequently, GPx reduces H_2_O_2_ to oxygen and water, similar to catalase. Additionally, GPx functions as a scavenger of lipid hydroperoxides (17).

Furthermore, a system of negative feedback is present between Nrf2 activation and the signaling pathway of extracellular signal-regulated kinase 1/2 (ERK1/2). ERK1/2 proteins belong to the mitogen-activated protein kinase (MAPK) family, which is associated with pro-inflammatory signaling pathways (20). Additionally, ERK-1/2 can phosphorylate and inhibit PPARγ (22). Considering these findings, it appears that any agent capable of activating the antioxidant pathway or inhibiting lipogenesis may be effective in the treatment or progression of NAFLD. Consequently, numerous studies have reported positive effects in improving the symptoms of steatohepatitis using agents such as saroglitazar, a PPARα/γ dual agonist, and curcumin (the principal constituent of turmeric), which is recognized for its antioxidative and anti-inflammatory characteristics (23, 24). 

Therefore, to explore and compare the mechanisms of action of curcumin and saroglitazar, we investigated the effects of these substances on non-alcoholic steatohepatitis induced in rats following a high-fat diet (HFD). The diet included the administration of a high-fat emulsion via gavage.

## Materials and Methods

To induce NAFLD, we prepared a high-fat emulsion following the methodology outlined in the studies conducted by Zou *et al*. (10). This emulsion, containing 4243 kcal/L of energy, comprised 77% fat, 9% carbohydrates, and 14% protein, and was fortified by vitamin/mineral supplements. It was kept at 4 °C and warmed to 42 °C prior to administration.

***Medicines***

Saroglitazar was acquired from Cadila Healthcare Limited, located in Ahmedabad, India. Additionally, curcumin powder with 98% purity (98% curcuminoids) was obtained from Abidi Pharmaceutical Company in Tehran, Iran. These medications were then dissolved in a solution of 0.5% sodium carboxymethyl cellulose (Na-CMC).

***Study design***

46 adult male Wistar rats, aged between 6 and 8 weeks and weighing 180–200 grams, were acquired from Ahvaz Jundishapur University of Medical Sciences’ Experimental Animal Center. These rats underwent a 7-day period of adaptation in a controlled environment with a steady temperature of 25.3 °C, relative humidity of 58%, and a light/dark cycle lasting 12 hr each. Then, in order to induce NAFLD, 36 rats were randomly selected and fed HFD (10 ml/kg oral emulsion) for 6 weeks as the high-fat group and received 30 mg/kg streptozotocin (STZ) by IP injection at the end of the 6th week; Another 10 rats, as the normal control group (NC), received a standard food diet. Biopsies were taken from two rats in the NC group and four rats in the high-fat group to evaluate the development of NAFLD or NASH after a 6-week period.

Group 1 (or NC) was given a solution containing 0.5% Na-CMC. The second group was fed HFD at a daily dose of 10 mg/kg. The third group received HFD along with saroglitazar at a dose of 3 mg/kg per day (24). The fourth group was also fed HFD and administered curcumin at a dose of 200 mg/kg daily (25). The fifth group, similarly fed HFD, was treated with a combination of saroglitazar and curcumin.

After a six-week treatment period, rats were sacrificed by intraperitoneal administration of a high dose of a ketamine and xylazine cocktail while fasting for 12 hr. Blood samples were then collected from the aorta, and serum was obtained by centrifugation at 4500 rpm for 15 min. This serum was preserved at -20 °C until biochemical tests were performed. The liver index was calculated by removing the liver tissues, washing them in chilled saline, drying them, and then weighing them. These liver tissues were then divided into two parts: one was fixed in 10% formalin for histopathological studies, and the other was stored at -80 °C for subsequent gene expression studies.

***Biochemical measurements***

All measurements were performed on fresh frozen serum. Serum lipoprotein levels (mmol/l) (Low-density lipoprotein (LDL-C) and high-density lipoprotein (HDL)) and liver enzymes (IU/L) (alanine aminotransferase (ALT) and aspartate aminotransferase (AST)) were evaluated using routine laboratory methods.

***Gene expression analysis***

Frozen liver tissue was used for RNA extraction. The RNA kit (Yekta Tajhiz, Iran) was used to isolate total RNA. cDNA was produced using the the PrimeScript RT reagent kit (Amplicon, USA) based on reverse transcription. The produced cDNA was then used to analyze a quantitative real-time polymerase chain reaction (RT-PCR) by an ABI StepOnePlus RT-PCR system. According to the planned cycle schedule, the real-time PCR reaction was carried out. The sequence of designed primers is mentioned in Table 1. The ΔCt method was used to compare the expression of each gene to that of the glyceraldehyde-3-phosphate dehydrogenase (GAPDH) as the internal control gene. The fold-change value was calculated using the 2^(-ΔΔCt) formula. This value represents the quantity changes of the target group compared to the corresponding control group.

***ROS production assay***

ROS levels in liver tissue were evaluated using a commercially available kit (KiaZist, Iran). Briefly, ROSs in the sample oxidize the Fe^2+^-o-dianisidine complex to a Fe^3+^ which forms a colored complex with xylenol orange that can be detected spectrophotometrically at a wavelength of 560 nm, and its intensity is proportional to ROS levels. The assay is calibrated with H_2_O_2_, and the results are reported as nmol H_2_O_2_ equivalent/ml.

***Histopathological evaluations***

Liver tissue samples were fixed in 10% buffered formalin for histological analysis for 24 hr. Samples were embedded in paraffin wax after dehydration using gradient alcohol. Slices were cut from the paraffin blocks (6–7 μm) and placed on poly-L-lysine-coated slides. Next, using xylene and graded ethanol, the slices were deparaffinized and hydrated. Hematoxylin-eosin (H&E) staining solutions were used to stain these sections, and then the slides were observed and histologically evaluated using a light microscope.

***Western blot***

The ERK1/2 and Nrf2 protein levels were measured using western blot analysis. Liver tissue was homogenized, and proteins were extracted using RIPA lysis buffer and a protease inhibitor cocktail. The total protein content was then measured with the Bradford assay, and an equivalent amount of protein was loaded onto a 10% sodium dodecyl sulfate-polyacrylamide gel electrophoresis (SDS-PAGE) gel, which was then transferred onto a PVDF membrane. The membranes were blocked with 5% skim milk for 1 hr at (20–25 °C). Overnight incubation at 4 °C was performed with primary antibodies targeting phosphorylated ERK1/2 and Nrf2 proteins (1:1000, Santa Cruz). After three washes with PBS-Tween20, the membranes were incubated with an HRP-conjugated secondary antibody for 1 hr. After additional washes, the bands were detected using the Enhanced Chemiluminescence (ECL) Detection Kit (Amersham Corp., Arlington Heights, IL, USA). Protein amounts were normalized against GAPDH levels and semi-quantified using ImageJ software.

***Statistical analysis***

The SPSS software (v.21, IBM Corporation) was used to perform statistical analysis. T-tests were employed to assess the results of variable experiments between the HFD and control groups. For comparisons involving more than two groups, one-way analysis of variance (ANOVA) with a Tukey-Kramer *post hoc* test was utilized. The data were shown as mean ± standard deviation (SD), and statistical significance was set at *P*<0.05.

**Figure 1:**
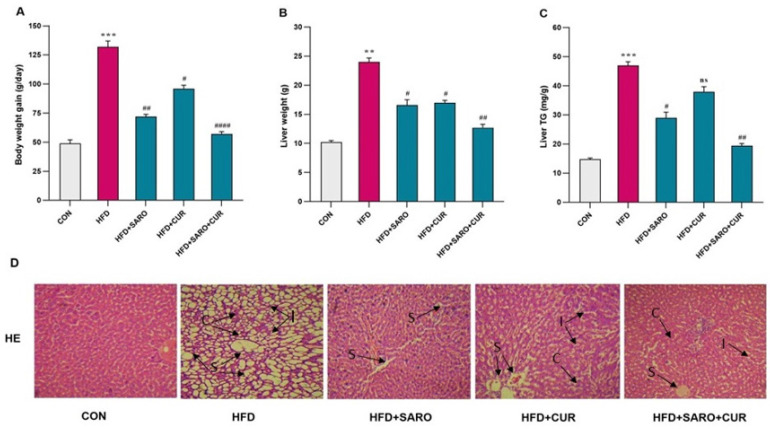
Assessment of body weight, liver index, TG levels, and morphology

The high-fat diet (HFD) fed rats showed significant increases in body weight (A), liver index (liver weight/body weight ratio) (B), and liver Triglyceride levels (C) compared to the control group. After drug administration, these variables decreased significantly compared to the HFD group. Histopathological evaluations of liver tissue were shown by hematoxylin-eosin staining (D). SARO = Saroglitazar; CUR= curcumin, CON= control, (C: congestion; I: inflammation; S: steatosis). Statistical significance between groups is shown by the * and # symbols. (*** *P*<0.001), (# *P*<0.05), (## *P*<0.01), and (### *P*<0.001).

**Figure 2:**
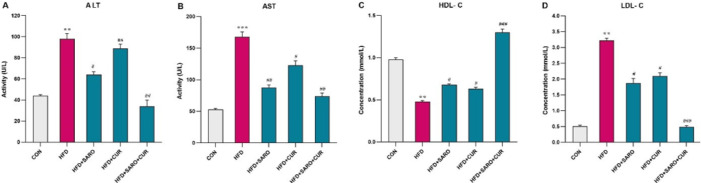
Assessment of the serum levels of lipid profiles and liver enzymes

AST, ALT, and LDL-C levels show a significant increase, and HDL-C levels show a significant decrease in high-fat diet (HFD) fed rats. These variables changed significantly in the drug-treated groups. SARO= Saroglitazar; CUR= curcumin, CON= control. Statistical significance between groups is shown by the * and # symbols. (*(*P*<0.05), (** *P*<0.01), (# *P*<0.05), (## *P*<0.01), and (### *P*<0.001).

**Figure 3:**
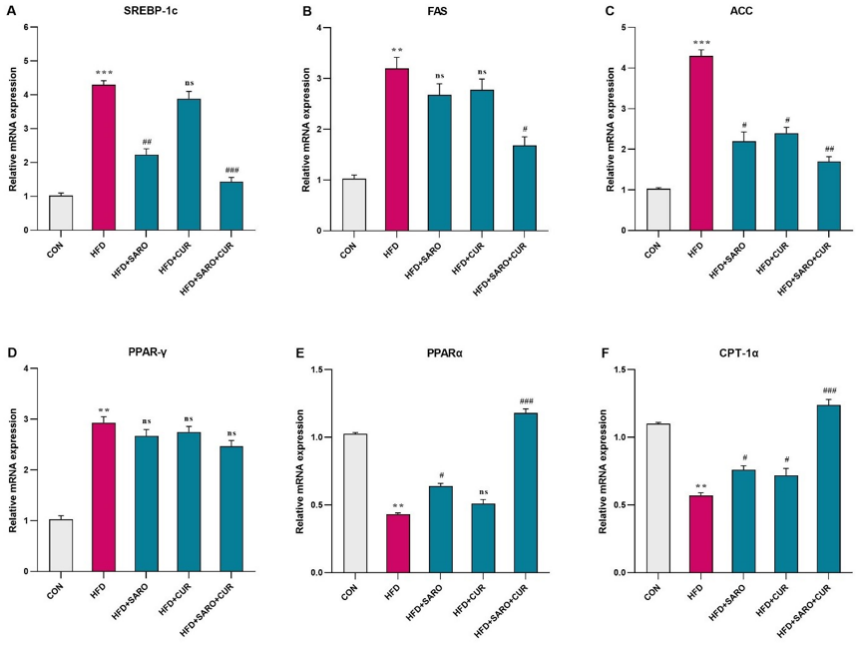
Assessment of the level of lipid metabolism genes in liver tissue of rats fed a high-fat diet (HFD)

The relative expression levels of SREBP-1c, FAS, ACC, PPARγ, PPARα, and CPT-1α mRNA were assessed using real-time PCR. The mRNA expression each gene was normalized to GAPDH expression (internal control). In each sample pair, the fold change was obtained utilizing 2^-ΔΔCT^ formula. SARO= Saroglitazar; CUR= curcumin, CON= control. Statistical significance between groups is shown by the * and # symbols. (**P<0.01), (*** *P*<0.001), (#*P*<0.05), (##*P*<0.01), and (###*P*<0.001).

**Figure 4:**
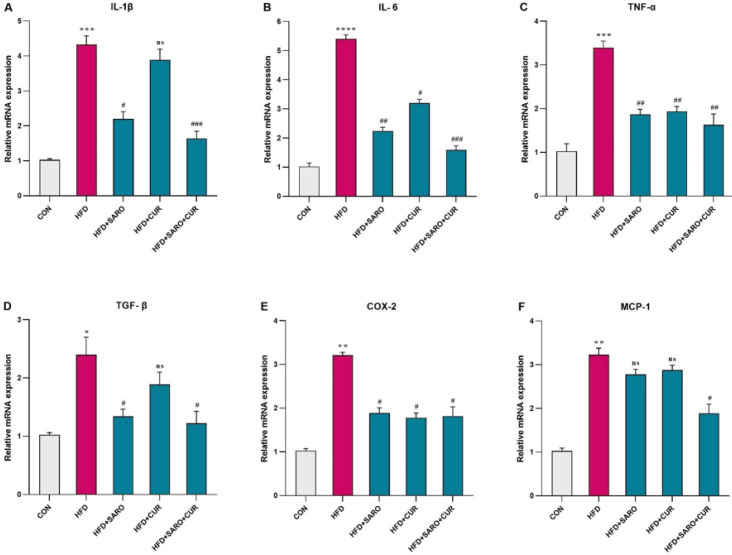
Assessment of the level of pro-inflammatory cytokines in liver tissue of rats fed a high-fat diet (HFD)

The relative expression of IL-1, IL-6, TNF-α, TGF- β1, COX-2, and MCP-1 mRNA were assessed using real-time PCR. The mRNA expression each gene was normalized to GAPDH expression (internal control). In each sample pair, the fold change was obtained utilizing 2^-ΔΔCT^ formula. SARO= Saroglitazar; CUR= curcumin, CON= control. Statistical significance between groups is shown by the * and # symbols. (* *P*<0.05), (**P<0.01), (*** *P*<0.001), (# *P*<0.05), (## *P*<0.01), and (### *P*<0.001).

**Figure 5:**
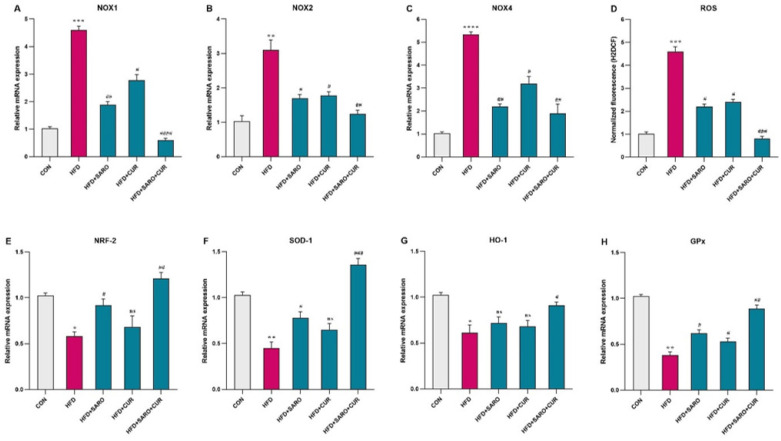
Assessment of antioxidant defense in liver tissue of rats fed a high-fat diet (HFD)

The expression levels of Nrf2, SOD-1, HO-1, GPx, and NOX1, 2, 4, mRNA were assessed using real-time PCR. The mRNA each gene was normalized to GAPDH expression (internal control). In each sample pair, the fold change was obtained utilizing 2^-ΔΔCT^ formula. (D) shows the levels of reactive oxygen species (ROS) before and after drug treatment. SARO= Saroglitazar; CUR= curcumin, CON= control. Statistical significance between groups is shown by the * and # symbols. (* *P*<0.05), (** *P*<0.01), (*** *P*<0.001), (# (*P*<0.05), (## *P*<0.01), and (### *P*<0.001).

**Figure 6:**
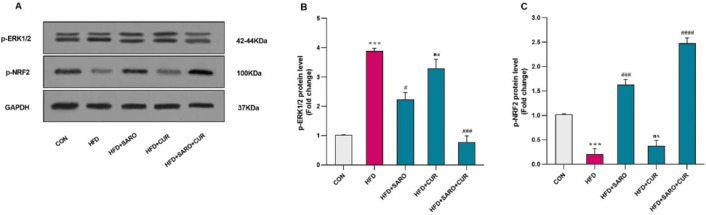
Western blotting detection for ERK-1/2 and Nrf2 protein expression

(A) bands of western blotting in different groups of rats fed a high-fat diet (HFD) compared to control group. (B, C) the proteins expression of ERK-1/2 and Nrf2 were estimated by determining intensities of ERK-1/2 and Nrf2 bands relative to GAPDH bands using ImageJ software. SARO= Saroglitazar; CUR= curcumin, CON= control, Nrf2= nuclear factor erythroid 2-related factor 2, ERK1/2= extracellular signal-regulated kinase 1/2.. Statistical significance between groups is shown by the * and # symbols. (*** *P*<0.001), (## *P*<0.01), and (### *P*<0.001).

**Figure 7:**
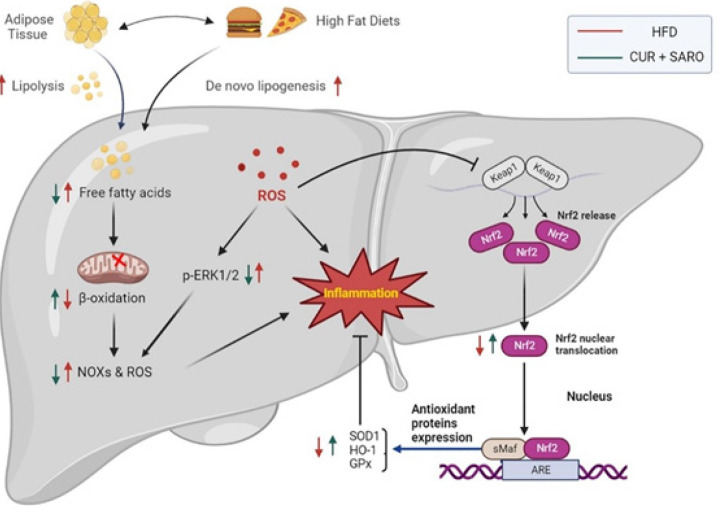
Mechanism of action of saroglitazar and curcumin in improving NAFLD is that both components regulate lipid metabolism and inflammation through nuclear factor erythroid 2-related factor 2 (Nrf2) and extracellular signal-regulated kinase 1/2 (ERK1/2)

## Results

***Effect of curcumin and saroglitazar on steatohepatitis, body mass, and liver index***

After eight weeks, the group on HFD showed a significant increase in body weight, liver index (liver weight/body weight ratio), and liver triglycerides (*P*<0.05) compared to the control group. Following drug treatment, significant changes were observed in these variables in comparison to the HFD group (Figure 1A, B, C). Figure 1D displays the hematoxylin-eosin staining of tissue sections from five experimental groups. The liver tissue from the HFD group, as stained by hematoxylin-eosin, exhibited microvesicular and macrovesicular steatosis. In contrast, the control group displayed normal liver histology. Rats on HFD treated with curcumin + saroglitazar showed improvement in the histopathological features of non-alcoholic steatohepatitis (NASH). There was also a significant histological difference between the HFD group and the groups treated with HFD + curcumin and HFD + saroglitazar.

***Serum levels of lipid profiles and liver enzymes***

In the group receiving HFD, the levels of AST, ALT, and LDL-C were significantly increased, and the level of HDL-C was significantly decreased when compared with the NC group (*P*<0.05). Treatment with saroglitazar, curcumin, or their combination led to a significant decrease in AST, ALT, and LDL-C levels, and a significant increase in HDL-C levels when compared with the group fed HFD (*P*<0.05). The concurrent use of saroglitazar and curcumin demonstrated more pronounced effects than using either agent alone, as depicted in Figure 2.

***Expression of genes related to lipid metabolism***

The levels of SREBP1c, FAS, ACC, and PPARγ expression are significantly higher, and PPARα and CPT-1α expression is significantly lower, in comparison to the control group. After drug treatment, SREBP1c, FAS, ACC, and PPARγ expression decreased, while the mRNA levels of PPARα and CPT-1α increased compared to the HFD group (Figure 3).

***Expression of pro-inflammatory genes in liver tissue***

The inflammatory status of liver tissue was evaluated by quantifying the levels of pro-inflammatory cytokines IL-1, IL-6, TNF-α, TGF-β1, TGF-1, cyclooxygenase-2 (COX-2), and monocyte chemoattractant protein-1 (MCP-1). These cytokines were significantly higher in the group fed HFD compared to the group on a normal diet (Figure 4). Subsequently, a significant decrease in the expression of these cytokines was seen in the groups that received drug treatment (*P*<0.05).

***Expression of genes related to antioxidant defense***

To evaluate the oxidative status in liver tissue, we measured the relative expression of Nrf2, SOD-1, HO-1, and GPx as antioxidant enzymes and the expression of NOX1, 2, and 4 which are involved in ROS production. The expression of NOX1, 2, 4 was significantly higher in the HFD group compared to the control group (Figure 5), while the expression of antioxidant enzymes was significantly lower. Drug treatment with saroglitazar and curcumin significantly enhanced the expression level of antioxidant enzymes and significantly decreased the level of ROS.

***Comparison of protein expression level of ERK-1/2 and Nrf2 in different groups***

The levels of ERK-1/2 and Nrf2 proteins were estimated via the Western blot assay. The level of ERK-1/2 protein was significantly higher, while the level of Nrf2 protein was significantly lower in the HFD group compared to the control group (*P*<0.05) (Figure 6). Treatment with saroglitazar and curcumin significantly decreased the expression of ERK-1/2 and significantly enhanced the protein expression of Nrf2 in comparison to the high-fat group (*P*<0.05).

## Discussion

NAFLD is a chronic disorder characterized by steatohepatitis, capable of progressing to cirrhosis and hepatocellular carcinoma. Although NAFLD is a multifactorial disease, several studies have shown that insulin resistance and hepatic steatosis lead to ROS production and the establishment of an OS status, which is the main factor in the initiation and development of NAFLD (26).

Excessive consumption of fat in the diet increases the deposition of TG in the liver and causes steatosis. Also, increased free fatty acids (FFAs) induce fatty acid β-oxidation and subsequently overproduction of ROS. ROS leads to overexpression of SREBP1, which in turn up-regulates lipogenesis-related genes and worsens steatosis (26, 27). In agreement with this hypothesis, in the current study, a rat model of NAFLD was induced through administering HFD (HFD). Our findings demonstrated a significant increase in the levels of PPARγ, SREBP1, FAS, ACC, and ROS in the group receiving HFD compared to the control group. As demonstrated in other studies, our finding showed that ROS and the subsequent oxidative stress significantly reduced the levels of antioxidant enzymes (SOD-1, HO-1, and GPx), while liver enzyme levels (ALT and AST) increased significantly due to liver tissue damage (7). Furthermore, a significant increase was observed in body weight, liver index, and serum levels of LDL-c in high-fat-fed rats. 

On the other hand, mRNA levels of pro-inflammatory genes (IL-1, IL-6, TNF-α, TGF- β1, COX-2, and MCP-1) were also significantly increased due to excessive ROS production. Histological evaluation also confirmed NAFLD in the liver tissues of the HFD group.

After a six-week treatment with saroglitazar and curcumin, the rats demonstrated a significant decrease in ROS levels and pro-inflammatory cytokine expression compared to the HFD group. Additionally, there was an overexpression of antioxidant enzymes (SOD-1, HO-1). The levels of lipogenesis genes and serum ALT, AST, and LDL-C were also significantly reduced with saroglitazar and curcumin. A notable improvement in liver index and pathological changes was found in the drug-treated group. In agreement with our results, Akbari *et al*. demonstrated that saroglitazar ameliorates hepatic steatosis, reduces serum ALT and AST levels, and down-regulates the level of pro-inflammatory cytokines in the HFD-induced NASH group (24). Researchers showed that curcumin is an effective treatment for hepatic steatosis in individuals with NAFLD (28), and numerous studies have already shown the anti-inflammatory properties of curcumin (29).

To investigate the mechanism of action of saroglitazar and curcumin in improving NAFLD, we detected the expression of PPARs, SREBP1, Nrf2, and ERK1/2 in the liver tissues of NAFLD model rats. PPARs regulate several physiological processes, including lipid metabolism, oxidative stress, etc. (3, 30). Nrf2 is involved in resistance to OS (18) and ERK1/2 in the pro-inflammatory signaling pathway. So that there is a negative feedback loop between NRF2 activation and ERK1/2 (31). The results of investigating the mechanism (Figure 7) showed that saroglitazar and curcumin treatment caused overexpression of PPARα in the NAFLD model, which is the upstream negative regulator of SREBP1. Therefore, as expected, a decrease in the expression of lipogenesis genes (FAS, ACC) was observed.

Our findings also showed that these compounds were able to increase the expression and phosphorylation of Nfr2, which is the upstream positive regulator of SOD, GPx, HO-1, and NOXs, and improve the OS status. Although studies have indicated that PPARγ is a target gene for Nfr2 (17, 19). We also discovered that the level of PPARγ was significantly decreased in the liver tissue of the NAFLD model, while conversely, Nrf2 and PPARα expression were significantly increased. This may be attributed to the tissue type or a result of the suppression of SREBP1 by PPARα, an inducer of PPARγ expression (16). On the other hand, administration of saroglitazar and curcumin significantly reduced the level of pro-inflammatory cytokines (IL-1, IL-6, TNF-α, TGF- β1, COX-2, and MCP-1) that were enhanced abnormally in the liver tissues of NAFLD. This is due to the interaction between OS and inflammation (31); when ROS decreases, inflammation also subsides. Many studies have demonstrated that Nrf2 reduces inflammation by not only modulating reactive oxygen species (ROS) but also by negatively controlling NF-κB. NF-κB, in turn, controls the level of pro-inflammatory factors (32).

Besides, in the liver tissue of the treated rats, ERK1/2 expression and phosphorylation were significantly decreased. The decrease in ERK1/2 protein can be attributed to the increased expression of Nfr2. Similarly, other studies have demonstrated that increased Nfr2 expression can significantly inhibit Erk1/2 activation, which is a pro-inflammatory signal. Meanwhile, Erk1/2 activation can also suppress Nrf2 activity (31). Furthermore, researchers have reported that the phosphorylation of ERK1/2 (active form) and dephosphorylation of ERK1/2 decrease and increase PPARα expression, respectively (33). Hence, ERK1/2, like Nrf2, indirectly regulates lipid metabolism.

Generally, we observed that saroglitazar and curcumin regulated the expression and activation of Nrf2 and ERK1/2 proteins. Furthermore, these compounds improved disturbed lipid metabolism in the NAFLD model by decreasing the gene expression associated with lipid synthesis and increasing the gene expression linked to fatty acid β-oxidation. Moreover, these compounds increased antioxidant capacity and reduced ROS levels. In addition, we demonstrated that the combined pharmacological effects of saroglitazar and curcumin are more effective than either alone. However, our rat model, induced by HFD, may not fully reflect the complexity of human NAFLD, and extrapolating these results to clinical scenarios requires caution. Hence, further research is necessary to comprehensively understand the complex signaling pathways.

To develop effective therapeutic approaches, future research is recommended to include the conduct of clinical trials on human subjects, optimization of dosage and combination ratios, further elucidation of molecular mechanisms, and exploration of synergistic effects with other therapeutic agents. Additionally, comparisons with standard treatments will be important to determine the relative effectiveness of saroglitazar and curcumin.

## Conclusion

This study demonstrates that saroglitazar, a PPARα/γ dual agonist, and curcumin (the principal constituent of turmeric), can be effective in the treatment of NAFLD. This efficacy is attributed to their ability to activate the Nrf2 pathway and suppress the ERK1/2 signaling pathway, thereby inhibiting lipogenesis and increasing antioxidant capacity. Our findings also show that saroglitazar and curcumin have synergistic effects, and it is suggested that curcumin, being a natural compound, could serve as a suitable substitute for saroglitazar.

## Authors’ Contributions

A KH conceived and supervised the study. R A designed the experiments. R A and N D performed all experiments. G HO contributed to disease diagnosis and selection of patients. B JA analyzed the data. R A and N D wrote the first draft. N D, R A, and H A revised the manuscript. A KH contributed to interpreting the results. All authors read and approved the final manuscript.

## Conflicts of Interest

None.
